# Emotional burnout in psychiatrists from different regions of Ukraine during the war

**DOI:** 10.1192/j.eurpsy.2026.11844

**Published:** 2026-07-31

**Authors:** N. O. Maruta, T. V. Panko, V. Y. Fedchenko, I. O. Yavdak, O. E. Semikina

**Affiliations:** Borderline Psychiatry, “Institute of Neurology, Psychiatry and Narcology named after P. V. Voloshyn of NAMS of Ukraine” SI, Kharkiv, Ukraine

## Abstract

**Introduction:**

The war in Ukraine has led to an increased burden on the medical system and on medical workers, which increases the risk of burnout. A special group in this regard is psychiatrists.

**Objectives:**

To determine emotional burnout among psychiatrists in Ukraine in wartime.

**Methods:**

210 psychiatrists from different regions of Ukraine were surveyed using the “Questionnaire of socio-demographic indicators in mental health workers to identify emotional burnout during war”, the burnout questionnaire by K. Maslach and S. Jackson. The study was conducted on the REDCap platform.

**Results:**

The survey data indicate changes in working conditions during the war: worsening psychological working conditions (41.43% of respondents), increased tension in working conditions (79.04%), increased workload (59.52%), and worsening financial support for the organization of the work process (20.95%). Only 8.57% of respondents noted that the conditions and nature of work had not changed. According to the results of the K. Maslach and S. Jackson scale, it was found that 93.35% of respondents had manifestations of burnout syndrome. According to the “Emotional Exhaustion” scale, 65.71% of psychiatrists had a high level of emotional exhaustion - 29.07 points; 27.62% had an average level of 12.64 points; only 6.67% had a low level - 5.64 points. On the “Depersonalization” scale, the highest indicators were observed in 40.00% of the surveyed, 17.33 points, average indicators in 27.62%, 7.40 points, and low indicators in 32.38% of people (1.75 points). On the scale “Reduction of personal achievements” low indicators were established in most cases - 39.77 points in 78.10% of cases, average indicators occurred in 13.81% of people - 26.72 points, high indicators were in 8.10% of people - 15.82 points, which reflects a tendency to negatively assess one’s competence and productivity, a decrease in professional motivation, an increase in negativism towards official duties, a tendency to absolve oneself of responsibility, a tendency to isolate oneself from others, a tendency to avoid participation in work.

**Conclusions:**

The results of the study indicate that 93.35% of the surveyed psychiatrists had signs of burnout (of varying degrees of severity and different stages of formation): the initial stage of burnout was observed in 18.09% of the surveyed, the stage of stress/emotional exhaustion - in 33.33%, the stage of pronounced burnout - in 37.15%, the final stage - in 4.76%. The data obtained reflect the need to develop a system of preventive measures to prevent the development of burnout syndrome in psychiatrists during the war.

**Disclosure of Interest:**

None Declared

